# Supplementary material for: Thermal Evolutions to Glass-Ceramics Bearing Calcium Tungstate Crystals in Borate Glasses Doped with Photoluminescent Eu3+ Ions
Source: Materials (Basel). 2021 Feb 18;14(4):952. doi: 10.3390/ma14040952 (PMC7922195; doi:10.3390/ma14040952)
Supplement: Supplementary file 1 [file materials-14-00952-s001.pdf]

*Supplementary*

# Thermal Evolutions to Glass-Ceramics Bearing Calcium Tungstate Crystals in Borate Glasses Doped with Photoluminescent $Eu^{3+}$ Ions

**Takahito Otsuka [1], Martin Brehl [2], Maria Rita Cicconi [2], Dominique de Ligny [2] and Tomokatsu Hayakawa [1,3,*]**

1. Field of Advanced Ceramics, Department of Life Science and Applied Chemistry, Graduate School of Engineering, Nagoya Institute of Technology, Gokiso, Showa, Nagoya, Aichi 466-8555, Japan; t.otsuka.098@stn.nitech.ac.jp
2. Institute of Glass and Ceramics, Department of Materials Science and Engineering, University of Erlangen-Nuremberg, Martensstraße 5, DE-91058 Erlangen, Germany; martin.brehl@fau.de (M.B.); maria.rita.cicconi@fau.de (M.R.C.); dominique.de.ligny@fau.de (D.d.L.)
3. Frontier Research Institute for Materials Science (FRIMS), Nagoya Institute of Technology, Gokiso, Showa, Nagoya, Aichi 466-8555, Japan

* Correspondence: hayatomo@nitech.ac.jp

**Raman spectra of the samples heated**

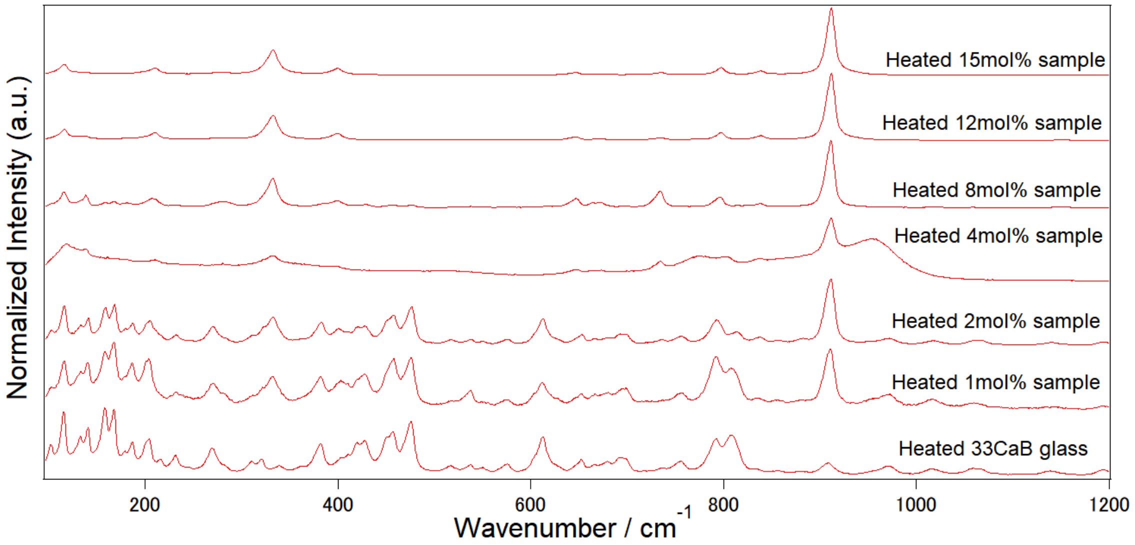


**Figure S1.** Raman spectra of the (100−*x*) ($33CaO$-$67B_2O_3$) − *x* $Ca_3WO_6$ (*x* = 0–15) glass samples at crystalline part after the post heat-treatment.

Figure S1 show Raman spectra of (100 − *x*) ($33CaO$-$67B_2O_3$) − *x* $Ca_3WO_6$ (*x* = 0–15) glass samples heat-treated at the condition given in Table 1 in the maintext. Raman peaks observed in *x* = 1–15 samples corresponding to 912, 838, 401, 336 and 218cm$^{-1}$ are assigned as $CaWO_4$ vibrations $\nu_1$ ($A_g$), $\nu_3$ ($B_g$), $\nu_4$ ($B_g$), $\nu_2$ ($A_g$) and $A_g$. The fact that the Raman spectrum of 4mol% sample includes a signal from the glass component implies a small amount of existence of the crystalized part in the glass sample.

**Photo image of ($33CaO$-$67B_2O_3$) − *x* $Ca_3WO_6$ glass-ceramics**

The heat-treatment (the detail for the sample preparation was found in the maintext) was applied for (100 − *x*) ($33CaO$-$67B_2O_3$) − *x* $Ca_3WO_6$ glasses. With the higher $Ca_3WO_6$ content the glasses were well crystallized, as shown in Figure S2.

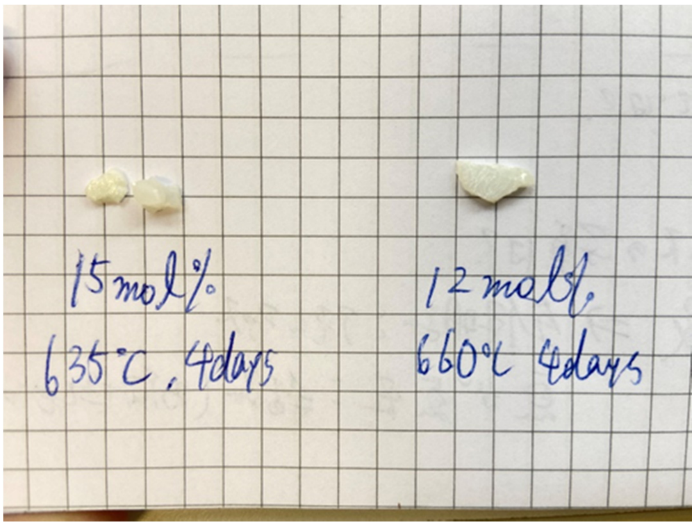


**Figure S2.** Photo-image of (100 − *x*) (33CaO-67$B_2O_3$) – *x* $Ca_3WO_6$ (*x* = 12 and 15 mol%) after the heat-treatment given in Table 1 in the maintext.

**PL and PLE spectra of $Eu^{3+}$ ions in glass/glass-ceramic and $CaWO_4$ crystal**

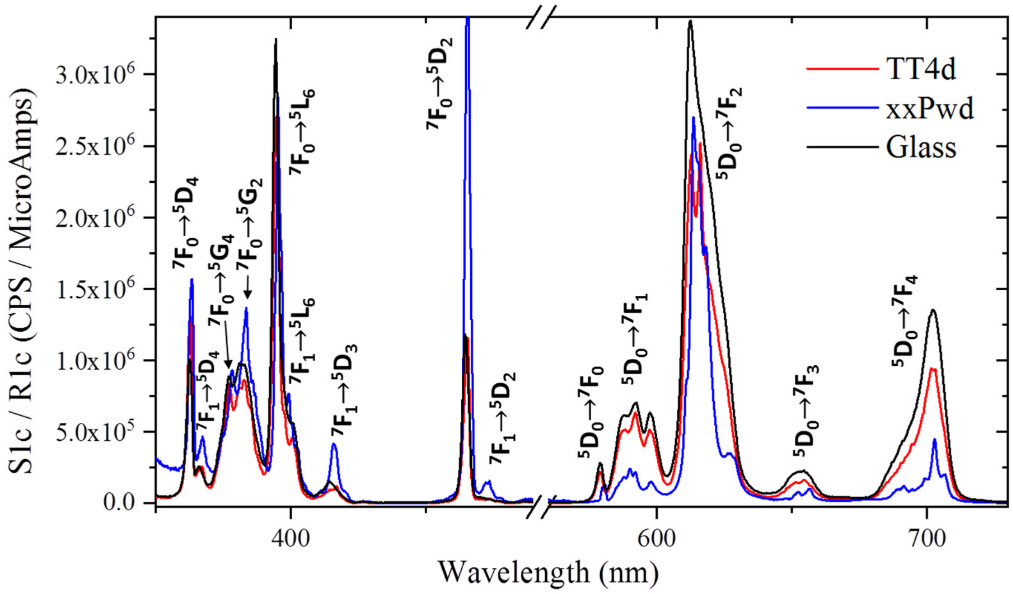


**Figure S3.** PL and PLE spectra of glass (Glass), glass-ceramic (TT4d) for the 15 mol% $Ca_{2.98}Eu_{0.02}WO_6$ samples, and $Ca_{0.98}Eu_{0.02}WO_4$ crystal (xxPwd). The glass-ceramic was composed of $CaWO_4$ crystals. The assignments of the optical transitions were given in the figure according to the literature (W.T.Canall, P.R.Fild, and K.Rajnak, “Electronic Energy Levels of the Trivalent Lanthanide Aquo Ions. IV. $Eu^{3+}$”, J.Chem.Phys. 49(10) (1968) 4450–4455).

The strong feature of $^7F_0$-$^5D_2$ transition in PLE spectra indicated the enhanced transition probability due to the electric dipole of $Eu^{3+}$ ions in the crystal sample, which coincides with the increased asymmetry ratio Λ = 7.5 of the crystal in conmarison with those of glass/glass-ceramic (See also the maintext).

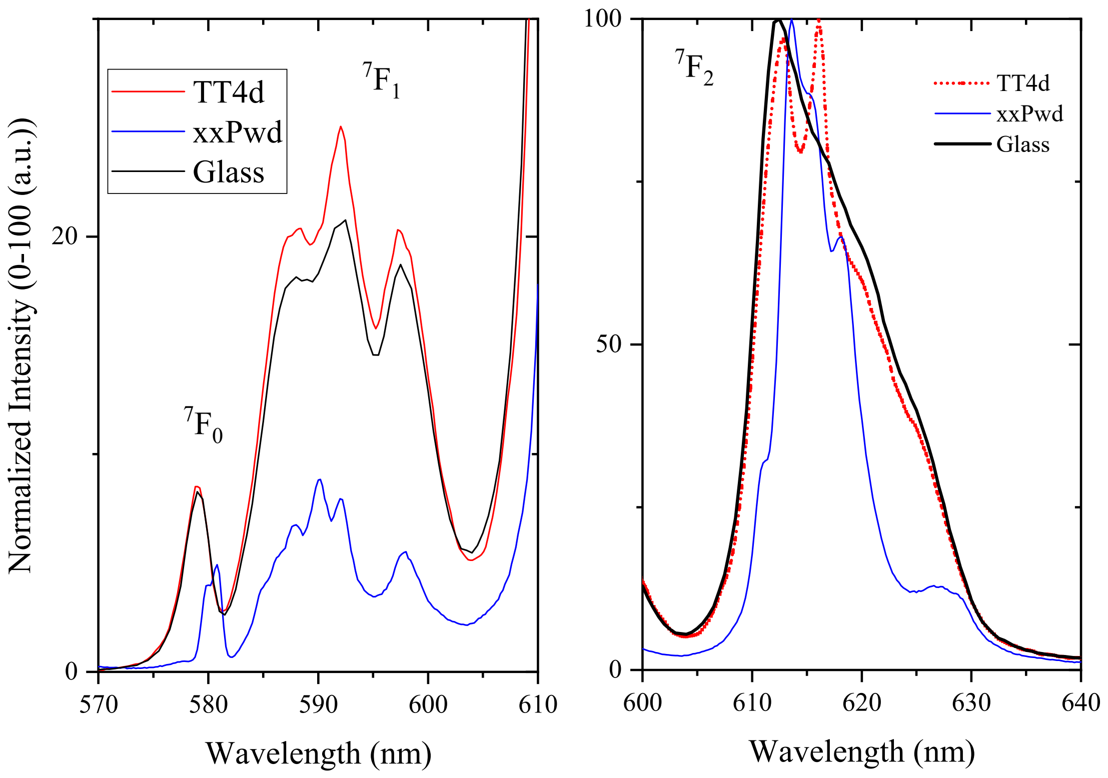


**Figure S4.** Enlarged PL spectra of $^5D_0$-$^7F_1$ and $^5D_0$-$^7F_2$ transitions normalized by 613 nm PL intensity for the glass (Glass), glass-ceramic (TT4d) for the 15 mol% $Ca_{2.98}Eu_{0.02}WO_6$ samples, and $Ca_{0.98}Eu_{0.02}WO_4$ crystal (xxPwd).

Figure S4 was obtained by a relatively high-resolution condition $\Delta\lambda$ = 0.5 nm with narrower widths of Entrance and Exit slits and a grating of 1200 gr/mm of monochromator used. A sharp feature of the $^5D_0$-$^7F_2$ PL spectrum of the $Eu^{3+}$ doped glass-ceramic indicates that $Eu^{3+}$ ions partially entered in the $CaWO_4$ crystals but still the majority of the ions are in the glass matrix. The reported PL decay lifetime of the $Eu^{3+}$ doped glass-ceramic was slightly decreased from the lifetime of $Eu^{3+}$-doped glass, and the PL decay curves were almost identical (See Figure 9 in the maintext). The observation coincides with the result given from Figure S4. The PL intensity ratio between $^5D_0$-$^7F_1$ and $^5D_0$-$^7F_2$ was confirmed to be almost the same for the glass and glass-ceramic but the $Eu^{3+}$-doped $CaWO_4$ crystals had a higher $^5D_0$-$^7F_2$ intensity. The asymmetry ratios for three samples were given in Figure 8 in the maintext. This makes sure that the local symmetry of $Eu^{3+}$ ions in the crystal powder is completely different.
